# *In vitro* antioxidant potential of *dicliptera roxburghiana*

**DOI:** 10.1186/1472-6882-13-140

**Published:** 2013-06-19

**Authors:** Bushra Ahmad, Muhammad Rashid Khan, Naseer Ali Shah, Rahmat Ali Khan

**Affiliations:** 1 Department of Biochemistry, Faculty of Biological Sciences, Quaid-i-Azam University, Islamabad 45320, Pakistan; 2 Department of Biotechnology, Faculty of Biological Sciences, University of Science and Technology, Bannu, Khyber Pakhtunkhwa, Pakistan

**Keywords:** *Dicliptera roxburghiana*, Lipid peroxidation, Total flavonoids, Antioxidants

## Abstract

**Background:**

Stress caused by free radicals accumulation result into many hazardous diseases. A number of investigations are focusing to find out the plant oriented natural antioxidant moieties. The basic aim of this research was to investigate the antioxidant potential, total Phenolic and flavonoids contents and photochemical screening of the crude methanol extract and its derived various fractions *Dicliptera roxburghiana* of Acanthaceae family.

**Methods:**

Crude methanol extract of aerial parts of *Dicliptera roxburghiana* (DRME) was partitioned in to *n*-hexane (DRHF), chloroform (DRCF), ethyl acetate (DREF), *n*-butanol (DRBF) and the remaining soluble portion as residual aqueous fraction (DRAF). We evaluated the antioxidant activities of the extract and various fractions through different analytical methods such as DPPH, superoxide anion, ABTS, H_2_O_2_, hydroxyl radical and phosphomolybdate radical inhibition. *In vitro* lipid peroxidation and reducing power of the plant was also analyzed. Total flavonoid and phenolic contents of the extract and all fractions were also quantified. Plant was also subjected for preliminary phytochemical screening to confirm the presence or absence of various constituents in the plant.

**Results:**

Phytochemical screening confirmed the presence of flavonoids, phenolics, tannins, alkaloids, saponins, terpenoids and coumarines. Quantitative analysis revealed the maximum amount of total phenolic and flavonoid contents in DRME while lowest in DRHF. Methanol extract, DREF, DRCF and DRBF exhibited promising antioxidant potential for DPPH, ABTS, H_2_O_2_, phosphomolybdate, superoxide anion and hydroxyl radical scavenging capabilities, while these were not appreciable for DRHF and DRAF. All fractions except DRHF and DRAF possess strong reducing power ability and showed appreciable lipid peroxidation inhibition.

**Conclusion:**

These research investigations revealed that *Dicliptera roxburghiana* is a potent source of natural antioxidants. Hence the plant can be used for management of different stress and anxiety related ailments.

## Background

Biochemical and physiological course of action, taking place nearly in all type of the living cells, result into production of harmful free radicals and reactive oxygen species [1]. These free radicals and reactive oxygen species damage the bimolecular moieties such as DNA, proteins and lipids; ultimately become leading source of different chronic serious ailments like cancer, aging, diabetes, atherosclerosis etc. [2]. To overcome this hazard nature has provided us a defense shield in the form of dietary antioxidants from plants. Medicinal plants play a crucial role for the management of various ailments [3-8]. Plants are richly supplied with vitamins, flavonoids, coumarins, phenolics, terpenoids, tannins and alkaloids etc. that are strong antioxidants [9]. Hence, medicinal plants contain many key compounds that can be used for the management of oxidative stress induced diseases [10,11]. The positive outcome by intake of antioxidant moieties of plant origin have been publicized in a number of investigational and epidemiological studies [12,13].

*Dicliptera roxburghiana*, belongs to the family Acanthaceae, is a perennial herb with 2–7 dm long stems. Leaves are green and are slightly paler on lower surface with 1–3.5 cm long petioles. Flowers are arranged in axillary cymes and all bracts are short-villous especially along the margins, calyx lobes are of unequal size, 5–7 mm long; color of corolla varies from rose to purple. Capsules are 6–7 mm long [14]. Saturated fatty acids (C-15 to C-31) and flavonoids (apigenin, kaempferol, luteolin and apigenin-7-O-glucoside) were isolated and identified from *Dicliptera roxburghiana*[15]. Powder of the plant is used as general tonic [16], for wound healing [17] and is non toxic [18]. As there is no published data for the antioxidant potential of *D*. *roxburghiana*, so this study was designed to investigate the antioxidant potential of the plant along with its total phenolic and flavonoid contents.

## Methods

### Plant material

*Dicliptera roxburghiana* was collected at maturity from the campus of Quaid-i-Azam University in April 2012. Identification of plant was validated by Dr. Mir Ajab Khan, Department of Plant Sciences Quaid-i-Azam University, Islamabad and a voucher specimen (accession#125521) was submitted in the Herbarium of Pakistan situated at Quaid-i-Azam University Islamabad, Pakistan. It was shade dried (28 ± 2°C) and leaves were coarsely pulverized to obtain dry powder in Willy Mill to 60-mesh size. Powder was stored at room temperature for further analysis.

### Preparation of methanol extract

Methanol was selected as extraction solvent due to its ability to dissolve a vast variety of compounds in it. Plant powder (2.0 kg) was soaked in crude methanol (4.0 L) in large container and was regularly shaken for five days at room temperature (28 ± 2°C). Than it was filtered through Whatmann filter paper No. 45 and the re-extraction of the residue was repeated twice. Filtrate was dried under rotary vacuum evaporator (Panchun Scientific Co, Kaohsiung, Taiwan) at 40°C to yield concentrated dry extract. Methanol extract of plant *D*. *roxburghiana* (DRME) yielded 200 g dark green viscous material (10%) that was stored at 4°C for further investigations.

### Preparation of fractions

Further fractions were made by suspending 4 g of crude methanol extract in distilled water (200 ml). This solution was then successively partitioned with *n*-hexane, chloroform, ethyle acetate and *n*-butanol in separating funnel and yielded DRHF (4.7%), DRCF (4.2%), DREF (5.8%), DRBF (6.7%) respectively with residual aqueous fraction, DRAF (8.6%). Each fraction was collected, dried and stored at 4°C [19].

### Chemicals

All the chemicals used in these assays were of high polarity (99%). Ascorbic acid, gallic acid, rutin, Folin-Ciocalteu’s phenol reagent, AlCl_3_.6H_2_O, 2,2-Diphenyl-1-Picrylhydrazyl (DPPH), 2,2- azino-bis (3-ethylbanzthiazoline-6- sulphonic acid (ABTS), potassium oxidopersulphate, ammonium molybdate, phenazine methosulphate (PMS), nitroblue tetrazolium (NBT), ferric chloride, potassium chloride, trichloroacetic acid (TCA), thiobarbituric acid (TBA), potassium ferricynide, Mayer’s reagent, FeCl_3_ were purchased from Sigma Co. (St. Louis, MO, USA). H_2_SO_4_, 2-deoxyribose riboflavin, Na_2_CO_3_, NaOH, NaNO_2_, H_2_O_2_ were purchased from Wako Co. (Osaka, Japan). All analytical grade solvents e.g. *n*-hexane, chloroform, ethyl acetate and *n*-butanol were used with 99.8% purity level and were obtained from Merck Co. (Darmstadt, Germany). Ultrapure TM water purification system (Lotum Co., Ltd., Taipei, Taiwan) was used to get deionized distilled water.

### Quantitative analysis

#### Total polyphenolic contents quantification

Total polyphenolic contents were measured by spectrophotometric method according to Bursal and Gulcin [20]. Extract/fraction (1 ml) was mixed with 9 ml of distilled water. Folin-Ciocalteu’s phenol reagent (1 ml) was put in to the mixture followed by 7% Na_2_CO_3_ solution (10 ml) and was shaken. The mixture was diluted 25 times with deionized distilled water. After 90 min absorbance was measured at 750 nm. Gallic acid (0–100 mg/ml) was used for standard curve. The measured total polyphenolics were expressed as mg Gallic acid equivalents (GAE) per g of dried sample.

#### Total flavonoids contents quantification

Total flavonoid contents were analyzed by following the Protocol of Park et al. [21]. For this purpose 0.3 ml extract, 3.4 ml methanol (30%), 0.15 ml of NaNO_2_ (0.5 M) and 0.1 ml of AlCl_3_.6H_2_O (0.3 M) were added in a test tube and mixed well. After 6 min 1 ml NaOH (1 M) was put into the mixture. The absorbance was checked at 506 nm. Rutin solution (0–100 mg/L) was used to form standard curve of total flavonoids. Total flavonoids were measured as mg of rutin equivalent/g of dried sample.

### Investigation of antioxidant potential

Each fraction (1 mg) was dissolved in 1 ml methanol to form stock solutions. Then further dilutions (20, 50, 100, 150, 200 and 250 μg/ml) of each fraction were formed.

### Diphenyl-1-Picrylhydrazyl (DPPH) assay

DPPH assay was performed according to the procedure of Brand-Willium et al. [22]. For this purpose 0.1 M DPPH (1, 1- diphenyl-2-picrylhydrazyl) solution was made in methanol and absorbance of the solution was adjusted at 0.95 at 515 nm. Sample (100 μl) was mixed with 1 ml DPPH solution and incubated at 37°C for 30 min. Methanol was used as control. After 30 min absorbance was noted at 515 nm. Ascorbic acid was used as standard. DPPH scavenging activity was calculated according to following formula and IC_50_ was calculated.

Inhibition (%) = [(Absorbance of control – Absorbance 

of sample) / Absorbance of control] × 100

### Azino-bis (3-ethylbanzthiazoline-6- sulphonic acid (ABTS) scavenging activity

Protocol of Re et al. [23] was adopted for evaluation of ABTS scavenging activity. ABTS solution (7 mM) was mixed with potassium oxidopersulphate (2.45 mM) solution and was placed in the dark for 12–16 h to get a dark colored ABTS working solution. The solution was diluted with 50% methanol and absorbance was adjusted at 0.7 (±0.02) at 734 nm. Sample (100 μl) was mixed with 1 ml of ABTS working solution and decrease in absorbance was read 1 min after adding the sample and then up to 6 min. Percentage inhibition was calculated according to following formula

Inhibition (%) = [(Absorbance of control – Absorbance 

of sample)/ Absorbance of control] × 100.

### Phosphomolybdate assay

This assay was performed according to the procedure of Umamaheswari and Chatterjee [24]. Working reagent was formed by mixing 0.6 M H_2_SO_4_, 28 mM sodium phosphate and 4 mM ammonium molybdate. Sample (100 μl) was mixed with 1 ml working reagent to form mixture which was placed in water bath for 90 min at 95°C. After cooling the mixture at room temperature absorbance was read at 765 nm against a blank. Ascorbic acid was run as standard. Antioxidant activity was determined according to following formula:

Antioxidant effect (%) = [(control absorbance-sample

 absorbance) / (control absorbance)] × 100.

### Superoxide anion radical scavenging assay

Protocol of Beauchamp and Fridovich [25] was followed to investigate the above mentioned assay. Phosphate buffer 0.5 ml (50 mM, pH 7.6), 0.3 ml riboflavin (50 mM), 0.25 ml PMS (20 mM) and 0.1 ml NBT (0.5 mM) were mixed to make reaction solution. Sample (100 μl) was mixed with 1 ml reaction solution and mixture was placed under fluorescent lamp for 20 min. Absorbance was measured at 560 nm. Ascorbic acid was taken as standard. The percent inhibition of superoxide anion generation was calculated by following formula:

Scavenging activity (%) = (1- absorbance of sample / 

absorbance of control) × 100.

### Hydrogen peroxide scavenging activity

This activity was assessed by following the procedure of Ruch et al. [26]. Hydrogen peroxide solution (2 mM) was formed in phosphate buffer (50 mM, pH 7.4). Sample (100 μl) was mixed with 400 μl phosphate buffer and 600 μl of H_2_O_2_. Reaction solution was vortexed and incubated for 10 min at room temperature and then absorbance was analyzed at 230 nm. H_2_O_2_ scavenging activity was calculated by following formula:

Hydrogen peroxide scavenging activity = (1- absorb-

ance of sample/absorbance of control) × 100.

### Hydroxyl radical scavenging assay

This activity was determined by following the protocol of Halliwell and Gutteridge [27]. Reaction solution consisted of 2- deoxyribose 500 ml (2.8 mM) in phosphate buffer (50 mM, pH 7.4), 200 ml premixed ferric chloride (100 mM) solution (1:1; v/v), 100 ml H_2_O_2_ (200 mM) and extract solution (100 ml). Ascorbate 100 ml (300 mM) was added to the reaction solution and incubated for 1 h at 37°C. TBA solution 1 ml (1%; w/v in 50 mM NaOH) and 1 ml TCA (2.8%; w/v aqueous solution) were added to the reaction solution. Reaction solution was heated in boiling water bath for 15 min and then was allowed to cool. Absorbance was noted at 532 nm and scavenging activity of hydroxyl radical was calculated as follow:

Scavenging activity (%) = (1- absorbance of sample / 

absorbance of control) × 100.

### *In vitro* lipid peroxidation assay at chicken liver

A healthy chicken was killed; liver was removed and was washed with 0.9% saline. The fresh liver tissue was homogenized in buffer, pH 7.4 (0.174 M KCl and 0.25 mM Tris HCl) Hunter et al. [28]. The study procedure for the animal care and experimentation was permitted by Ethical Committee of Quaid-i-Azam University Islamabad.

Lipid peroxidation assay was performed by following the protocol of Iqbal et al. [29]. Phosphate buffer 0.58 ml (0.1 M; PH 7.4), 200 μl sample, 200 μl liver homogenate and 20 μl ferric chloride (100 mM) were combined to form mixture which was placed in a shaking water bath for 1 h at 37°C. Reaction was terminated by adding 1 ml TCA (10%). TBA 1 ml (0.67%) was added to all the tubes which were placed in boiling water bath for 20 min. Then test tubes were shifted to crushed ice bath and were centrifuged at 2500 × g for 10 min. Absorbance of the supernatant was checked at 535 nm and was calculated as nM MDA/min/mg tissue by using molar extinction coefficient of 1.56 × 10^5^ /M/cm.

### Reducing power assay

Reducing power assay was investigated by following the protocol of Gulcin et al. [30]. Phosphate buffer 2 ml (0.2 M, pH 6.6), sample 2 ml, and 2 ml potassium ferricynide (10 mg/ml) were mixed and were incubated for 20 min at 50°C. After incubation 2 ml TCA (100 mg/ml) was added into the mixture. This mixture (2 ml) was mixed with 2 ml distilled water and 0.4 ml ferric chloride (0.1%; w/v) and absorbance was noted at 700 nm after 10 min. Increase in absorbance was noted that was an indication of strong reducing power capability.

### Preliminary phytochemical screening

Preliminary phytochemical screening of *D*. *roxburghiana* was done by different qualitative assay procedures to validate the presence or absence of flavonoids, phenolics, coumarines, alkaloids, tannins, saponins, phlobatannins, anthraquinone and terpenoids.

### Alkaloid screening

Alkaloidal screening was done according to the protocol of Farnsworth and Euler [31] with some modifications. Extract (500 mg) was moistened with Ca(OH)_2_ solution (40%) to remove acids, phenolics and tannins. Further extraction was carried out with the chloroform (10 ml) twice and filtrate was concentrated. Aqueous acid solution was added to the concentrated extract to form alkloidal salts which were soluble in aqueous layer and impurities in the organic phase were separated. Aqueous phase was treated with ammonia solution to precipitate the alkloidal salts which were alkaline to litmus paper. Extract was mixed with Dragendroff’s reagent. Dark orangish red color determined the presence of alkaloids.

### Saponins screening

Plant saponins were detected according to the procedure of Wall et al. [32]. Blood standardization was done to get red blood cell suspension. One ml extract was added in 10 ml of standardized blood suspension and was kept at room temperature (28 ± 2°C) for 5 min. Complete hemolysis of red blood cells was considered an indication of presence of saponins.

### Terpenoids screening

An amount of 5 mg sample was mixed with 5 ml distilled water and 2 ml of chloroform was put into the mixture along with subsequent addition of 3 ml H_2_SO_4_. A reddish brown line was formed that was a sign of presence of terpenoids [33].

### Coumarins screening

Sample (300 mg) was taken in a test tube that was covered with filter paper moistened with NaOH (1 N). Test tubes were kept in a boiling water bath for few min. After that filter paper was analyzed under UV light and presence of coumarins was confirmed as yellow fluorescence [34]. Another confirmatory test was carried out; filter paper was sprayed with phenylboric acid, β-aminoethyl ester [35] and the presence of coumarins was confirmed.

### Flavonoid screening

Sample (25 mg) was put into 50 ml distilled water and was filtered. 10 ml of filtrate was combined with 5 ml of dilute ammonia solution after that few drops of concentrated H_2_SO_4_ were added. Yellow color was a sign of presence of flavonoids [36].

### Tannins screening

50 mg sample was put into 20 ml distilled water and was filtered. Few drops of FeCl_3_ were combined with filtrate and presence of tannins was confirmed by appearance of brownish green color [36]. Filtrate paper was also sprayed with two drops of basic lead acetate solution. Appearance of white precipitate confirmed the presence of tannins [37].

### Statistical analysis

Results are analyzed as mean ± SD from triplicate observations. *In vitro* antioxidant assays were analyzed by ANOVA test followed by Tukey’test (P < 0.05) to find out the significant differences among IC_50_ of different fractions in each assay. Graph Pad prism software was applied to determine the IC_50_ values.

## Results

### Preliminary phytochemical screening

Preliminary phytochemical screening of all derived fractions of *D*. *roxburghiana* demonstrated the presence of flavonoids, phenolics, terpenoids, tannins, alkaloids, saponins and coumarins as shown in Table 1.

**Table 1:** **Preliminary phytochemical screening of *D***. ***roxburghiana***

**Extract**	**Flavonoids**	**Phenolics**	**Alkaloids**	**Tannins**	**Saponins**	**Coumarins**	**Terpenoids**
**DRME**	+	+	+	+	+	+	+
**DRHF**	+	_	+	+	+	_	+
**DRCF**	+	+	_	+	+	_	+
**DREF**	+	+	+	+	_	+	+
**DRBF**	+	+	+	+	+	+	+
**DRAF**	+	+	_	+	+	+	+

*DRME D*. *roxburghiana* methanol extract, *DRHF D*. *roxburghiana n*-hexane fraction, *DRCF D*. *roxburghiana* chlorofrorm fraction, *DREF D*. *roxburghiana* ethyl acetate fraction, *DRBF D*. *roxburghiana n*-butanol fraction, *DRAF D*. *roxburghiana* aqueous fraction.

### Total phenolics and flavonoid contents and extraction yield

Extraction yield of the methanol extract and various fractions ranged from 4.2 ± 3.6% to10.3 ± 1.1% with an ascending order of DRCF < DRHF < DREF < DRBF < DRAF < DRME (Table 2). Maximum yield with methanol extraction determined highest quantities of extractable compounds whereas chloroform extraction yield was very low.

**Table 2:** **Extraction yield**, **total phenolic and flavonoid contents of *D***. ***roxburghiana***

**Plant extract**	**Total phenolics**	**Total flavonoids**	**Extraction yield**
**DRME**	189.4 ± 1.1^a^	235.3 ± 0.8^a^	10.3 ± 1.1^a^
**DRHF**	13.5 ± 0.9^f^	18.7 ± 1.2^d^	4.7 ± 5.7^e^
**DREF**	163.3 ± 2.1^c^	213.3 ± 1.1^b^	5.8 ± 4.8^d^
**DRCF**	179.5 ± 0.3^b^	218.7 ± 1.5^b^	4.2 ± 3.6^e^
**DRBF**	135.9 ± 0.4^d^	203.3 ± 1.7^b^	6.7 ± 0.9^c^
**DRAF**	82.7 ± 0.5^e^	69.4 ± 0.8^c^	8.6 ± 2.5^b^

Values are represented as mean ± (n = 3), Different letter (^a-e^) represent significance (P < 0.05).

*DRME D*. *roxburghiana* methanol extract, *DRHF D*. *roxburghiana n*-hexane fraction, *DRCF D*. *roxburghiana* chlorofrorm fraction, *DREF D*. *roxburghiana* ethyl acetate fraction, *DRBF D*. *roxburghiana n*-butanol fraction, *DRAF D*. *roxburghiana* aqueous fraction.

Total phenolic contents of methanol and all other fractions of *D*. *roxburghiana* varied widely, ranging from 13.5 ± 0.9 mg to 189.4 ± 1.1 mg Gallic acid equivalent/g dry weight with the reference of standard curve (Y = 0.004_X_, r^2^ = 0.995). Total phenolic contents were solvent dependent. Highest phenolics contents were found in methanol extract whereas *n*-hexane fraction reflected small quantity of phenolics. Flavonoid contents also varied widely among different fractions. Methanol extract exhibited highest flavonoid contents whereas DRHF showed small amount, ranging from 235.3 ± 0.8 mg to 18.7 ± 1.2 mg Rutin equivalent/g dry weight with reference standard curve (Y = 0.003_X_, r^2^ = 0.932) as depicted in Table 2.

### *In vitro* antioxidant potential

*In vitro* antioxidant potential of the plant was determined using different analytical assays. All antioxidant assays provide considerable support to antioxidant prospective of plant in comparison with standard ascorbic acid.

### DPPH scavenging activity

**Figure 1:**
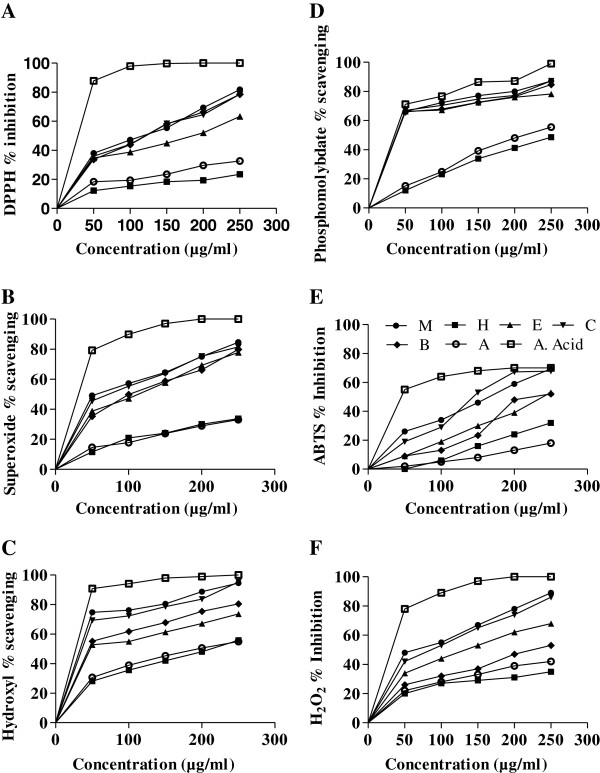
**Antioxidant activity of different fractions and methanol extract of *D*. *roxburghiana *at different concentrations with a mean ± SD (n = 3): (A) DPPH radical scavenging activity, (B) Superoxide radical scavenging activity, (C) Hydroxyl radical scavenging activity, (D) Phosphomolybedate radical scavenging activity, (E) ABTS radical scavenging activity and (F) Hydrogen peroxide radical scavenging activity.** M: methanol, H: *n*-hexane, E: ethyl acetate, C: chloroform, B: *n*-butanol, A: aqueous and A. acid: ascorbic acid.

Figure 1A describes the DPPH activity of the plant and was found in the following order: DRCF > DRME > DRBF > DREF > DRAF > DRHF. IC_50_ values were 114.27 ± 1.2 μg/ml, 121.03 ± 1.5 μg/ml and 124.23 ± 1.1 μg/ml for DRCF, DRME and DRBF respectively (Table 3). Although antioxidant effects of various extracts were low as compared to standards still they were appreciable. Positive correlations were existed among DPPH IC_50_ values of fractions and their total phenolics and flavonoid contents.

**Table 3:** **Antioxidant effect** (**IC**_**50**_) **of different fraction of *D***. ***roxburghiana***

**Plant extract**	**IC**_**50 **_**μg/ml**					
	**DPPH radical scavenging activity**	**Superoxide radical scavenging activity**	**Phosphomolybdate scavenging activity**	**Hydroxyl radical scavenging activity**	**ABTS radical scavenging activity**	**H**_**2**_**O**_**2 **_**radical scavenging activity**
**DRME**	121.03 ± 1.5^b^	56.46 ± 0.3^b^	24.18 ± 0.6^b^	17.94 ± 0.1^b^	153.78 ± 0.2^b^	77.38 ± 0.5^c^
**DRHF**	>250 ^d^	>250^e^	234.57 ± 0.3^f^	46.25 ± 0.7^e^	>250^e^	>250^f^
**DREF**	195.23 ± 0.4^c^	112.71 ± 0.6^d^	40.73 ± 0.2^d^	32.56 ± 0.9^d^	217.79 ± 0.6^d^	136.61 ± 0.9^d^
**DRCF**	114.27 ± 1.2^b^	73.18 ± 0.4^c^	35.81 ± 0.4^c^	23.62 ± 0.1^c^	123.75 ± 0.2^b^	88.59 ± 0.3^c^
**DRBF**	124.23 ± 1.1^b^	106.24 ± 0.7^d^	36.15 ± 0.5^c^	40.87 ± 0.5^e^	195.87 ± 0.3^c^	205.81 ± 0.2^e^
**DRAF**	>250^d^	>250 ^e^	188.26 ± 0.9^e^	67.24 ± 0.3^f^	>250^e^	>250^f^
**Ascorbic acid**	14.37 ± 0.9^a^	26.81 ± 0.4^a^	20.76 ± 0.3^a^	6.87 ± 0.4^a^	54.72 ± 0.3^a^	29.18 ± 0.6^b^

Values are represented as mean ± (n = 3), Different letter (^a- f^) represent significance (P < 0.05). *DRME D*. *roxburghiana* methanol extract, *DRHF D*. *roxburghiana n*-hexane fraction, *DRCF D*. *roxburghiana* chlorofrorm fraction, *DREF D*. *roxburghiana* ethyl acetate fraction, *DRBF D*. *roxburghiana n*-butanol fraction, *DRAF D*. *roxburghiana* aqueous fraction.

### Superoxide anion scavenging activity

Figure 1B reflects the superoxide radical quenching capability of various fractions of the plant and was found in the following order: DRME > DRCF > DRBF > DREF > DRAF > DRHF. IC_50_ values (Table 3) varied widely among different fractions and were appreciable for DRME (56.46 ± 0.3 μg/ml) and DRCF (73.18 ± 0.4 μg/ml). These values were low as compared to standards at the same dose but still were significant.

### Hydroxyl radical scavenging activity

All fractions exhibited a good percentage inhibition as described in Figure 1C and behave as potent radical scavengers. Methanol extract and DRCF showed strong antioxidant potential against this radical with IC_50_ values 17.94 ± 0.1 μg/ml and 23.62 ± 0.1 μg/ml respectively. IC_50_ of different fractions was in the following order: DRME > DRCF > DREF > DRBF > DRHF > DRAF.

### Phosphomolybdate radical scavenging activity

Methanol extract showed the highest percentage inhibition with IC_50_ value 24.18 ± 0.6 μg/ml when compared with IC_50_ value (20.76 ± 0.3 μg/ml) of standard ascorbic acid at the same dose level. Among all fractions IC_50_ values were significantly different (Table 3) and decrease in the following manner: DRME > DRCF > DRBF > DREF > DRAF > DRHF as shown in Figure 1D.

### ABTS radical scavenging activity

ABTS radical scavenging effects were significant (P < 0.05) among all fractions as shown in Table 3 and reflect strong antioxidant potential of the plant. Figure 1E describes inhibition percentage of methanol and all derived fractions of *D*. *roxburghiana*. ABTS antiradical activity decreases in the order of: DRCF > DRME > DRBF > DREF, with significantly different IC_50_ values ranging from 123.75 ± 0.2 to 217.79 ± 0.6 μg/ml.

### H_2_O_2_ radical scavenging activity

This radical scavenging activity was also concentration dependent with significant IC_50_ (P < 0.05) values as described in Table 3. All fractions reflect a good measure of antioxidant activity to scavenge HO radical (Figure 1F) except DRHF and DRAF. Order of activity decreased in given manner: DRME > DRCF > DREF > DRBF.

### Correlation between IC_50_ of antioxidant assays and total phenolic and flavonoid contents

A positive correlation was found for total phenolic contents and radical scavenging assays with significant R^2^ values (R^2^ = 0.883, 0.8588, 0.7154, 0.8635) for IC_50_ values of DPPH, superoxide, phosphomolybdate and ABTS radical scavenging activity respectively where as correlation of phenolic contents was weak for H_2_O_2_ and hydroxyl radical scavenging activity with R^2^ values 0.5158 and 0.5883 respectively (Table 4).

**Table 4:** **Correlation between IC**_**50 **_**values of antioxidant activities and flavonoids and phenolics contents of *D***. ***roxburghiana***

**Assays**	**Correlation R**^**2**^	
	**Flavonoids**	**Phenolics**
IC_50_ of DPPH radical scavenging activity	0.9634**	0.8830*
IC_50_ of Superoxide radical scavenging activity	0.9632***	0.8588**
IC_50_ of Phosphomolybdate radical scavenging activity	0.8924**	0.7154*
IC_50_ of ABTS radical scavenging activity	0.9458***	0.8635**
IC_50_ of H_2_O_2_ radical scavenging activity	0.3375	0.5158
IC_50_ of Hydroxyl radical scavenging activity	0.6608*	0.5883

Values are represented as mean ± (n = 3), *, **, *** shows level significance (P < 0.05; P < 0.01, P < 0.001).

For total flavonoid contents a significant correlation (R^2^ = 0.9634, 0.9632, 0.8924, 0.9458 and 0.6608) was found between total flavonoid contents and IC_50_ values of DDPH, superoxide, phosphomolybdate, ABTS and hydroxyl radical respectively (Table 4).

### Lipid peroxidation inhibition

**Figure 2:**
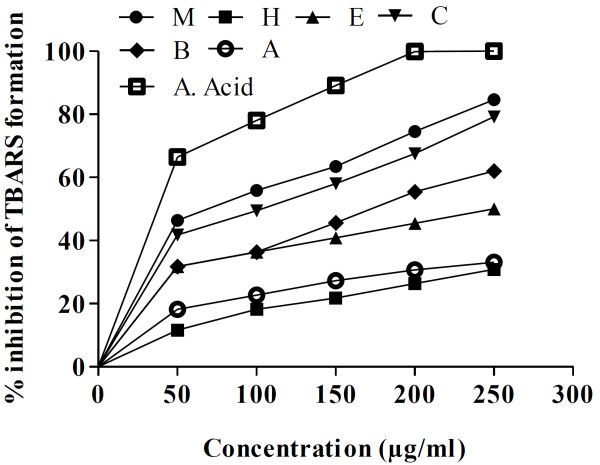
**Lipid per oxidation % inhibition of different fractions of *D*. *roxburghiana*.** M: methanol, H: *n*-hexane, E: ethyl acetate, C: chloroform, B: *n*-butanol, A: aqueous, G. Acid: gallic acid, A. Acid: ascorbic acid.

Figure 2 describes dose concentration curve for the % inhibition of lipid peroxidation of different plant derived fractions as compared to standard ascorbic acid. It was demonstrated that DRME, DRBF, and DRCF have appreciably reduced the TBARS contents formed during lipid peroxidation.

### Reducing power of *D*. *roxburghiana*

**Figure 3:**
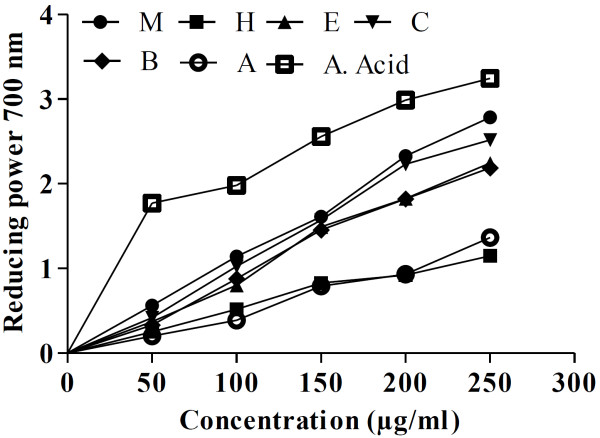
**Reducing power absorbance at 700 nm of different fractions of *D*. *roxburghiana *at different concentrations.** M: methanol, H: *n*-hexane, E: ethyl acetate, C: chloroform, B: *n*-butanol, A: aqueous, A. Acid: ascorbic acid.

As reducing power is a significant marker to measure the antioxidant capability so it was determined that increase in reducing power was actually the measure of antioxidant manifestations of the plant. Reducing power was also concentration dependant. Reducing power activity was not significant at low doses as compared to ascorbic acid but gradual increasing concentrations of fractions increased the reducing power capability of DRME, DRCF, DRBF and DREF significantly as demonstrated by Figure 3.

## Discussion

Free radicals play a key role in pathological manifestations. These phyto-originated constituents perform their role either by quenching the ROS or by acting as a defense shield to protect the antioxidant defense mechanism [24]. A number of techniques have been followed to evaluate the antioxidant potential of the plants [19] and it was confirmed that plant constituents are more secure than their synthetic counterparts [38,39].

Preliminary phytochemical screening of *Dicliptera roxburghiana* demonstrated the presence of nearly all active constituents of the plant such as flavonoids, phenolics, tannins, terpenoids, saponins, coumarins and alkaloids; explicate the antioxidant manifestations of the plant because a large number of investigations prove that plants are endowed with antiradical constituents which are powerful scavengers [40]. All these constituents have been shown to exhibit strong antioxidant scavenging activity for the radicals that are involved in the lipid peroxidation [10,41]. These active constituents of plants play a vital role in the treatment of different diseases such as tannins possess anti-inflammatory and anticancer activity [26,42]; flavonoids are antioxidant, anti-inflammatory any anticancer agents [43]; coumarins are antioxidant, maintain blood pressure and inhibit lipid peroxidation [44]; alkaloids possess antileukemic and anticancer activity [45] and saponin are antimicrobial agent and maintain the blood cholesterol level [46].

Extraction yield of different plant derived fractions varied widely; highest methanol extract yield proved that methanol behave as a good solvent for compound extraction where as DRCF yielded small quantity as compared to methanol and other solvents. Determination of flavonoid and phenolics contents determined that DRME and DRCF of *D*. *roxburghiana* possess strong antioxidant potential and this may be cumulative effect of both theses constituents. DRME exhibited highest phenolic contents where as contents obtained with DRHF were very low that is in accordance with other reports [47]. Plant flavonoid content determination also justified it to be a strong antioxidant source as such flavonoid rich plants could be a good source of antioxidant to inhibit pathological manifestations caused by free radical that are involved in lipid peroxidation pathways [48]. Our findings suggested that high phenolic and flavonoid contents of the plant are the major contributor to scavenge the free radicals in oxidation pathways.

Antioxidant scavenging capability of the plant has also revealed its strong antioxidant potential in quenching the radicals that cause oxidative trauma in cells. These scavenging activities were concentration dependent. DPPH, a purple colored bleaching solution, is an important source of free radical and is frequently used to measure the electron donating ability of the plant [49]. Extent of color change is proportional to the strength and concentration of antioxidant. In this study DRME, DRCF and DREF exhibited strong DPPH radical quenching activity that is positively correlated with their high phenolics and flavonoid contents. This was due to multifaceted nature of both these constituents that act as health promoting agents; neutralize the DPPH radical by donating hydrogen and protect from probable damage. Presence of apigenin and luteolin flavones in *D*. *roxburghiana* might contribute towards the DPPH radical scavenging activity.

Superoxide radical is the important oxygen radical among all reactive oxygen species [50]. Superoxide itself is a weak radical but may cause severe damage to the cell by generating hydroxyl radical and singlet oxygen [51]. Present study suggested that DRME and DRCF had potent scavenging activity against superoxide radical and a strong correlation exist with the flavonoid and phenolic contents of the plant. Present scavenging activity against superoxide radicals might be due to the luteolin because it possesses strong superoxide antiradical capability.

Another important radical called hydroxyl radical also play an important role in pathogenesis. Hydroxyl radical can easily bind to the polyunsaturated fatty acid of the cell membranes phospholipids and cause harmful effect to the cell [27]. Hydroxyl radical can easily bind to every molecule of the cell and cause mutation and carcinogenesis [52]. Hydroxyl radical was produced by the reaction of ferrous ion with 2-deoxyribose result into subsequent formation of malonaldehyde which was inhibited by TBA. Hydroxyl radical activity of the extracts is directly proportional to its antioxidant activity [53]. Structural relationship of apigenin, luteolin and kaempferol demonstrated the antiradical capacity for hydroxyl radicals. Depletion of hydroxyl radicals by the extract and fractions might involve the apigenin, luteolin and kaempferol flavonoids.

Phosphomolybdate is another assay that is performed to assess the overall antioxidant activity of the extract [54]. In the presence of antioxidant sample Mo (VI) reduced to Mo (V) with subsequent formation of green colored phosphomolybdenum V complex exhibiting maximum absorbance at 700 nm. Our findings demonstrate that *D*. *roxburghiana* has a very good antioxidant potential that was the collective contribution of phenolic and flavonoid components of the plant.

ABTS is a well known reactive radical that can lead to cell damage. In this assay ABTS oxidize to ABTS + chromophore on reaction with potassium persulphate; and reduced by antioxidant sample. Results justify that plant has ABTS radical scavenging activity and proved that plant may be used for the treatment of radical related stress appreciable due to ABTS radical quenching ability [10]. Loss of color in this experiment can be related with the reductive ability of various constituents; apigenin, luteolin and kaempferol in *D*. *roxburghiana*.

Hydrogen peroxide is detrimental reactive oxygen radical become toxic and damage the cell when converted into hydroxyl radical that may initiate lipid peroxidation and DNA mutations [55]. Present investigations suggested that all plant extracts were capable of quenching this radical that may be due to their phenolic contents that convert H_2_O_2_ to water.

Reducing power is another assay to measure the overall antioxidant prospective of extract. Plant antioxidants convert the Fe + 3/ ferric cyanide complex to ferrous form by contributing one electron with subsequent turning of yellow color reaction solution to green. Intensity of color change is proportional to the concentration of antioxidant present in test sample. Reducing capability can be monitored spectrophotometrically by increase in absorbance at 700 nm. Previous data reported that reducing ability actually responsible for antioxidant activity by donating hydrogen atom that in turn will break the fee radical chain [56]. Increase in absorbance by all fractions of the plant was an indication of strong antioxidant potential of *D*. *roxburghiana*.

Lipid peroxidation is another very important parameter to determine the total antioxidant potential of the plant. Lipids of cellular membranes are more susceptible to oxidative hazards. Lipid peroxidation by free radical results into the production of malonaldehyde (MDA) that reacts with DNA and cause mutation in the form of DNA adducts [57,58]. Active components of the *D*. *roxburghiana* inhibit the chain reaction to generate lipid peroxides. Present research design proved that all fractions of the plant significantly inhibited the lipid peroxidation that was due to the cumulative contribution of its phenolics and flavonoids contents that play important role in antioxidant manifestations.

## Conclusion

Natural product antioxidants significantly contribute in preventions of pathological consequences caused by free radicals. Furthermore plant derived antioxidant are safer and cheaper than their synthetic counterparts. We concluded from this research design that *D*. *roxburghiana* possesses strong antioxidant potential that may be due to the contribution of its phenolics and flavonoid contents and it would be advantageous to use the plant antioxidant in therapeutic drugs for the implications of human health.

## Competing interest

The authors declare that they have no competing interests.

## Authors’ contributions

BA made significant contribution to acquisition of data, analysis, drafting of the manuscript. MRK has made substantial contribution to conception and design, interpretation of data, drafting and revising the manuscript for intellectual content. NAS and RAK (Khan RA 0000-0003-0453-2090) participated in the design and collection of data and analysis. All authors read and approved the final manuscript.

## Pre-publication history

The pre-publication history for this paper can be accessed here:

http://www.biomedcentral.com/1472-6882/13/140/prepub
